# BUILDING OUR LARGEST DEMENTIA (BOLD) INFRASTRUCTURE FOR RISK REDUCTION

**DOI:** 10.1093/geroni/igac059.512

**Published:** 2022-12-20

**Authors:** Heidi Holt, Laura Whalen, Lisa Garbarino

**Affiliations:** CDC, Atlanta, Georgia, United States; CDC, Atlanta, Georgia, United States; Centers for Disease Control and Prevention, Atlanta, Georgia, United States

## Abstract

The Building Our Largest Dementia (BOLD) Infrastructure for Alzheimer's Act (PL115-406) supports public health agencies to develop a uniform dementia infrastructure across the US. Applying a robust public health approach to ADRD that emphasizes data driven decision-making and action along with primary, secondary, and tertiary prevention strategies (e.g., risk reduction, early diagnosis, linkages to treatment and care, and the prevention and management of comorbidities leading to preventable hospitalizations and poor health outcomes) for persons living with dementia and their caregivers. Recipients of the BOLD Public Health Programs funds are expanding jurisdiction Dementia coalitions, updating, or creating Dementia Strategic Plans, and implementing strategies from those plans that address a wide variety of life-course strategies for brain health and dementia, including risk reduction. This presentation will explain how risk reduction is integrated and provide examples of several activities being planned and implemented by recipients.

